# Thoughts upon Diagnosis, Pathology and Treatment*Published by direction of the Dallas Medical and Surgical Association. Read February 13, 1894.

**Published:** 1904-05

**Authors:** Henry K. Leake

**Affiliations:** Dallas, Texas


THE
### TEXAS MEDICAL JOURNAL.

ESTABLISHED JULY, 1885.

PUBLISHED MONTHLY.—SUBSCRIPTION $1.00 A YEAR.

VOL. XIX. AUSTIN, MAY, 1904. No. 11.

### Original Contributions.

For Texas Medical Journal.

### Thoughts Upon Diagnosis, Pathology and Treatment.*

BY HENRY K. LEAKE, A. M., M. D., DALLAS, TEXAS.

Considering the whole domain of medicine and to a large degree that of surgery, no matter how successful may be the aims of the physician, theory and inductive philosophy enter, more or less, into the problems that he is called upon to face and to attempt to solve. These methods of reasoning may appear either at the beginning of his task, or discover themselves when this is completed, but always leaving certain phases of the problem unexplained and demanding further investigation. Therefore, it seems that, whether we will or not, theory and inductive philosophy must be employed to suggest a plan for most of our work, or be constructed to account for much of the result of that work whether or not the latter be useful, or, in any way, prove satisfactory. From the intricate nature of physiology and pathology in their varying presentments owing to difference of type, inheritance and environments, often eluding the most searching investigation, it is not always the case that our results can be referred to manifest causes that stand out in bold relief from all other contributing factors. When analyzed, it may be found that the *causa morbi,* like a mixed infection, is composed of several elements, all of which exert an influence in the sum total of effect produced, and

*Published by direction of the Dallas Medical and Surgical Association. Read February 13, 1894.

our knowledge increases in proportion as we ascertain these several elements and give to each its relative value. It may be objected that, in the light of modern medicine and successful surgery, such uncertainty as regards diagnosis and treatment, at any rate, no longer exists in medicine to that extent that will justify this sweeping assertion. The germ causation of disease, which now seems to be the trend of all pathology, can be rigidly isolated and cultured, a systematic classification of etiology can be made and, in many cases, be predicated thereupon a sure remedy; thus we are afforded a convincing and trustworthy postulate in our diagnosis and therapeutics. Such a claim, however, will disappoint us to that degree as will make us often doubtful of our premises and conclusions.

It is true that the misleading tendencies of inductive philosophy, as applied to medicine in modern times, have been avoided somewhat in the promulgation of medical principles of etiology and treatment of disease, however convenient this philosophy has been in the past, but it must be acknowledged that this philosophy at present, either consciously or unconsciously, is far too much indulged by the practitioner. The "case fiend," who may be met on every corner, deceives himself and labors to impress us with his enthusiasm or superior judgment in formulating a general law for a rightful conception of disease from one, or it may be several, carelessly observed particulars. While it is not insisted that "*falsus in uno, falsus in omnibus*" should be a guiding principle in a medical creed, as well as in that of religion and morals, still the Baconian philosophy can not be rigidly applied to the solution of medical problems without great risk of delusion and disaster. The "case fiend" should withhold his persistent and often arrogant conceits until his experience in a certain line has been so large and directed by such a studious and trained intelligence that, at least, error may be severely minimized before a general law can be evolved that will arrange and correlate the several particulars and dignify the whole with eminent power and unvarying efficacy. But the "case fiend" is not to be tolerated in professional intercourse only, but intrudes his presence upon every page of medical journalism and in medical societies. "Most ignorant of that he is most assured," his authority may prove very brief if subjected to the searchlight of criticism that may dissolve his glassy essence that, like an ape, "Cuts such fantastic tricks before high heaven as make the angels weep."

Reckless theory and speculative philosophy long survived the Grecian and Roman schools of medicine, but medicine of the sixteenth century was impressed profoundly by the Baconian philosophy that invaded every branch of scientific thought which, reasoned out of the abstract, might be supposed to interpret and make useful a concrete emergency. Doubtless applying this philosophy to medical problems certain deductions were erected into what might be termed a canon of laws, numbers of which may be seen in every medical dictionary, but which, in these latter days, need only to be mentioned to be refuted, although some of them still maintain an important significance. These laws relate to questions chiefly of diagnosis and treatment; pathology also claims much of their import.

With respect to diagnosis, all laws and philosophy and inferentially experience are said often to be set aside by intuition—intuitive diagnosis—and this is asserted to be a quality of innate genius that "Soars above the common herd toiling up the steeps below." But there is no such thing as intuitive diagnosis, unless it be the product of that genius defined by Carlyle as having "an infinite capacity for painstaking;" the extraordinary intellect unconsciously employs inductive reasoning, successfully risks the well-observed albeit furtive glace of a pathognomic symptom, or a lightning-like analysis of the symptom-complex instantly may focus the mind upon its subject, but these mental operations, while the product of innate acuteness in determining medical questions, at all events, must have been more or less instructed by previous study and observation of disease through a rich and laborious experience. Lawson Tait was wont to make much of intuitive diagnosis, of which he was past master just as he was of his mechanical art, but it is well known that in his early life he had unrivaled opportunities with Simpson—himself a pre-eminent diagnostician and operator—and that the subsequent life of Tait increased constantly his wide clinical experience, and consequently his oft-repeated judgments. As he himself said, he "deliberated quickly;" an expression not so paradoxical as it may appear, if the observations made above be considered thoughtfully.

We must conclude that, at any stage of professional life, diagnosis by intuition is unsafe as a rule, haphazard and unscientific. There is no field of responsible effort where the scene so often varies, where rules and laws so often are defied, but where exceptions are ever ready to deceive our most hallowed experience, as that of medicine taken in its widest sense. It must be remembered that however well devised, theory will be based upon an hypothesis of which it is only "a clay model." Frequently it is employed in the realm of medicine with powerful effect by the trained intellect, it more rarely succeeds in the hands of the medical proletariat, but it is a dangerous tentative for those unequipped by nature and study with the mentality that will construct it upon the most rational and scientific grounds that may be defended by an appeal to ripe experience and intellectual development. Therefore, as Locke says, to judge properly is to differentiate a man of reason from a logical chicaner, although it may not be necessary to equip oneself wholly in the many particulars mentioned by this famouns sage.

Although these methods that influence us in our conception of diseases and manner of dealing with them may never be eliminated, as probably they could not without diminishing our hopes of scientific eminence attaining to that of the other sciences, we should safeguard our thoughts by considerations of prudence and wholesome scepticism, especially where dogmatism, prejudice, or thoughtfulness prevails to hazard statements that may entail not alone a limited, but widespread influence for evil. On reflection, we are amazed at the abject servility with which we bow to the opinions of others, even when probably opportunities are bestowed equally for arriving at the truth. Locke affirms: "All that he relies on without this perception, he takes upon trust upon the author's credit without any knowledge of it at all. This makes me not at all wonder to see some men so abound in citations and build so much upon authorities, it being the sole foundation on which they bottom most of their own tenets, so that in effect they have but a second-hand or implicit knowledge, i. e., are in the right if such a one from whom they borrowed it were in the right in that opinion they took from him, which indeed is no knowledge at all." Such a dependence upon authority may be excused in the young practitioner without experience who ruthlessly may not substitute his own opinions instead, but it does not absolve him from a studious examination of his author's logic that may be colored by haste, prejudice or interest. *"Nullius addictus jurare in verba magistri."*

Likewise this intellectual slavery may account for the formulating and apotheosizing the so-called banalities of medicine that often have traditional value as orthodox assumptions, but sometimes no foundation in fact whatsoever, and by which men have been tyrannized into routine grooves of thought not only in therapeutics, but in pathology and in symptomatology. Consequently, the protean and insidious manifestations of disease may deceive us into a false security, or stimulate an unwarranted therapeutic activity.

For example, gastric ulcer may progress to fatal perforation without antecedent pain or hemorrhage, or even apparent disturbance of the digestive function; rarely, however, in some form is the latter absent. Several years ago, a splendid woman under the care of Dr. Graham, of Dallas, died from gastric perforation that had been preceded by only vague symptoms of indigestion that previously were considered of little moment. Two similar cases were seen in consultation, although in the first the indigestion finally was emphasized just before the perforation occurred. Gastric carcinoma, also, may follow a like course. In medical literature will be found numbers of cases of this character that are rapidly accumulating.

According to Mayo,* a gall stone may lie ensconced within the common duct and may cause neither pain nor jaundice. Even its passage down the cystic or hepatic duct may be unattended by pain that shall much inconvenience the patient, yet death may ensue from peritonitis, cholangitis, or pancreatitis that now exploits its own supremacy.

In 1822, Bright determined the relation between dropsy and albuminuria. This was a startling and epoch-making discovery. Bright ascribed the albuminuria to insufficiency of the diseased kidney and portrayed several varieties of such disease, but one of which—contracted kidney—is now known to be a general arterial disease caused by perverted metabolism or by a special form of toxæmia. The classical sign of albuminuria, in these circumstances, may not appear, there being a mere trace of albumin in the urine, as often absent as present. Casts may not be discovered and the patient may die, the banal symptomatology being discredited throughout the entire history of the case. On the other hand, intermittent, or so-called cyclical albuminuria, may be present, particularly in young boys, the urine being normal in the morning and markedly albuminous in the evening, the condition extending over months, rebellious to treatment, yet the most painstaking scientific examination may fail to detect either kidney disease or constitutional causes to account for this curious abnormality. Moreover, it has been shown that albuminuria, in some way, may be related to business worries and irregular living. A few, or an isolated cast, now and then, may swim in the urine, but a healthy reform may reinstate the normal urinary output and the expectancy of life in no wise be curtailed. At the present time, I have under observation a patient of this class, and a

---

*Case reported by Brewer, *Medical Record*, January 23, 1904.

prominent physician of New York has predicted a long life for the individual whose urine is kept at a normal standard by a close supervision of diet and habits, there being in the system at large absolutely no symptom of aberrant physiology or pathological development.

Abscess of the liver has a banal symptomatology, but it is well known that exceptions are frequent. The brilliant and lamented Prof. E. S. Galliard, of Louisville, suffered for months with melancholia and other cerebral disturbance. Skillful physical diagnosticians, among whom was one with a deservedly national reputation, repeatedly examined the patient. There was neither pain, tenderness nor apparent enlargement of the liver. Hammond, of New York, believed that the cerebral derangement pointed to the liver. On exploring this organ, an abscess was discovered in the lobus Spigeleii, the patient dying a few days after a surgical operation. Dr. Samuel Egon, of Dallas, years ago had a very similar experience. He operated and proved his diagnosis that was based upon no better evidence than that presented by the case just mentioned.

In the category of medical "banalities," none stands more conspicuous than the symptomatology of ectopic gestation, but Dudley, of New York, declares that: "The symptoms and signs of extra-uterine preganancy are about as certain as steel stocks at the present time." It seems that in our conception of this malady, banal symptoms now must take second rank, or, if they are present, the significance is co-equal only with symptoms that may occur with other pelvic diseases.

Henceforward we shall be wary of the classical signs of appendicitis, for, as Bennett has demonstrated recently, in many cases after a more or less brief period, the pulse and temperature may drop to normal or even below, pain, tenderness, rigidity and distention about disappear, operation, now, may be declined, but the patient may be profoundly septic from an abscess of stinking pus surrounding the appendix. Moullin, in almost like circumstances, has found a gangrenous appendix. In these cases, the banal symptoms have disappeared, but others intrude themselves to establish the diagnosis with remarkable certainty.

In diphtheritic cases and other septic conditions, sudden death may occur from heart failure even when the patient is believed to be convalescing rapidly. Banal diagnosis of heart disease frequently made may be relied upon to decide the cardiac sufficiency. Autopsy may reveal the heart muscle far advanced in waxy and granular degeneration that gave no evidence of its presence during life by the ordinary examination, but the sphygmometer and other improved means in the future may warn us of existing danger in these cases. Many examples of common heart disease may be placed in the same category.

I operated on a patient who was invalided at home for a period of five weeks. His sickness began with agonizing pain exactly in the epigastrium and radiating towards the spleen. Several weeks previously he had experienced a slight pain with some tenderness over a small area in the left hypochondriac region. This discomfort was relieved by a small hypodermic of morphine. The next morning the man was at his business and actively followed this until a feeling of malaise decided him to remain at home. During the ensuing five weeks there was no symptom pointing to disease in the chest. His temperature was slightly elevated at night and his pulse seldom rose above 100. Cough and embarrassed respiration even on exertion were absent. There was no pain anywhere, nor was there the slightest dyspnœa. Decubitus was normal. There was some pallor of the skin, but no cyanosis. Apparently he could expand his chest to its full capacity without pain or inconvenience of any kind. Constipation and tympanitis were marked and troublesome features of the case. Physical examination of the chest was negative until the end of the third week, when signs of effusion appeared posteriorly, beginning at the base and rapidly extending up to the apex. This effusion made no alteration whatever in the subjective symptoms. Dr. Graham of Dallas was called and advised waiting. Ultimately a quart of very offensive pus was removed by operation. The symptom-complex of chest disease will be replaced often by pain, distension and rigidity in the abdomen that have misled the most astute diagnosticians and proved intractible to any method of treatment until the condition in the thorax is relieved. This is a common experience, a noteworthy illustration of which was the sensational case of Philip Sanger, a leading merchant of Dallas, who died several years ago. A patient of a confrere, supposed to be laboring under appendicitis, was operated upon, but the appendix was discovered by the surgeon to be in a normal condition. Subsequently a large purulent effusion was removed from the right pleural cavity. Such examples might be multiplied indefinitely to broaden our views,* revise our methods and sharpen our wits in dealing with the diagnostic and pathological subtleties that constantly beset us.

---

*"Barnard recently has drawn attention to the simulation of appendicitis by pleuro-pneumonic disease."

Probably there is no department of medicine where this reform is needed more urgently than current views of a pathology built up from certain well-known physiology of the nervous system —both cerebro-spinal and sympathetic. Reference here is to reflected pain as having its analogue in the normal reflexes, usually following the same paths, or, indeed, being heightened degrees of the normal beneficent impulse. The wide distribution, conjunction and interlacing of nerve ganglia and fibre impresses us with its evident, supported by its response to experimental, physiology. Nevertheless, orthodox views of such reflexes can not be claimed to be established infallibly. It is a question if nerve fibre is the sole medium of transmission of vital impulses. The cellular structure is one vast store-house of potential and reciprocal energy residing in the cells and in the electrons, of which these cells are composed. Metrosome, nay! atom is no longer to be taken as the unit of such energy, for "Now we know that the atom is in itself a tiny cosmos swarming with electrified particles traveling with immense speed in all directions;" not necessarily in specially confined or projected line of area, although in the main such action regulates the different functions of the economy, examples of which could not be cited without offense to any well-informed student of physiology. However, much of such postulate, probably, has been erroneously conceived and a physiological doctrine developed that is not bottomed on facts; or radium may show the relation of such energy to size and importance of cell structure and an inconceivably wide and constant diffusion of such energy, the special function of which we now can have only the faintest conception. The future, therefore, may alter radically our views of physiological reflex action, and consequently our diagnostic methods and treatment of disease, even in the domain of what is considered the most elementary functions of the body.

The arteries are kept tonus by the vaso-motor nerves, but Loeb has shown that a secretion from the supra-renal bodies must enter the blood current to stimulate and regulate the function, whether or not this be through nerve or musculature. The important function of the pancreas is evoked not by chyme stimulating nerve end-organs in the duodenum, but by "secretin," a product of digestion, that is absorbed and going the round of the circulation, reaches the pancreas to prove its true excitant.

Tait and Johnstone said that a small nerve ascended along the side of the uterus and ended in or near the horn. This was the "menstrual nerve" that must be constricted by the ligature if menstruation was to cease. Christopher Martin and others labored to show that there was a lumbar nerve centre that controlled the menstrual function, and thus, the "menstrual nerve," as well as the "ripened ovule" theory, was ignored, but the *tout ensemble* was not complete without nerve reflex action. Now! the ovary secretes an extract that is the *facile princeps* of the impulse that ends in the periodical congestion and desquamation of the endometrium.

If we consider pathology as physiology in abeyance, altered, or utterly wrecked, these considerations will indicate, not so much the direction in which our conceptions will be modified, but that changes surely will come as our knowledge expands from the studies of the future, notably in the domain of the nervous reflex system. In this field, reasoning by induction from local to remote pathology may be checked by acknowledging other factors *sui generis*, but all conjoined to produce the symptom-complex of disease. The latter presents oftenest in the form of vicious circles that may, or may not, be broken to relieve the patient, as when a painful ovary is removed with this purpose in view, or when pelvic disease of any character is treated for a like reason; but a vicious circle, if possible, must be demonstrable, otherwise either harm, or, at any rate, an unnecessary operation will follow. The various expressions of neurasthenia are conspicuous examples of such reasoning—a lacerated cervix has been sewed up for suboccipital pain, a tilted womb made a scape-goat for headaches and indigestion, while asthenopia, aural, and other cerebral conditions can be eliminated by the rapid dilator and curette; in many cases, certainly, but through fatal rupturing of the uterus, or by septicæmia. Pelvic disease with reflex symptoms has been treated for months, even years, when, as has been shown recently by Gilmore of Chicago and by myself, catarrhal enteritis is the *fons et origo mali*. A Socinus of pathology will yet arise to protest against this vicarious punishment of the sexual organs.

It is not denied that pathological reflex action does occur, but in the future our diagnosis of this symptom will be more discriminating. Whether or not we shall admit the dictum of Lasegue as expressed in his law that: "Superficial lesions or simple functional troubles of an organ increase the reflexes, while more or less pronounced organic lesions suppress them," is a question *sub judice*, but it is observed that reflex symptoms from pelvic disease are confined to the minor, which such major affections as ovarian tumors, fibroid tumors and malignant growths are seldom, if ever, said to cause them.

*Per contra* Byron Robinson conceived an elaborate scheme of pathological reflexes from pelvic disease, but Head, in his oft-quoted and valuable researches, insists that there are none such ascending to the cerebrum, at least from below the upper part of the rectum—the bladder, tubes and ovaries never giving rise to them. It appears that Head, by a series of experiments the most careful yet conducted, has discovered the pathways of nerve impulses for every portion of the body and along which pain is referred from diseased internal organs to the cutaneous surfaces. He is a modern "pathfinder" for the perplexed diagnostician. His teaching has been utilized in the diagnosis of chest, abdominal and pelvic disease. Regarding the first, the much discused lung-reflex experiments of Abrams may be referred to as corroborating proof of Head's researches. For several years past, I have tested the views of Head with the greatest satisfaction in the diagnosis of ovarian disease. The method is applied with practical results to the diagnosis of peritonitis and of appendicitis, as demonstrated by Moullin, Bishop, Sherren and other English surgeons, and recently by New York surgeons who, without giving credit to Head and Mackenzie, assert the diagnostic value of a principle that the latter were the first, in 1892, systematically to define and to emphasize. It is scientific and will be examined critically in all parts of the world, but will be established only by clinical experience that will not be supplanted by any kind of theory, philosophy or experiment whatever.

There are morality and conscience in accurate diagnosis, refined pathology and well-ordered treatment. The first two are the seed, the last is the flower in full bloom. If the seed be not sound, the leaves may turn to ashes in our hands.

